# Dissecting the proteome dynamics of the salt stress induced changes in the leaf of diploid and autotetraploid *Paulownia fortunei*

**DOI:** 10.1371/journal.pone.0181937

**Published:** 2017-07-27

**Authors:** Minjie Deng, Yanpeng Dong, Zhenli Zhao, Yongsheng Li, Guoqiang Fan

**Affiliations:** 1 College of Forestry, Henan Agricultural University, Zhengzhou, Henan, P.R. China; 2 Institute of Paulownia, Henan Agricultural University, Zhengzhou, Henan, P.R. China; Bhabha Atomic Research Centre, INDIA

## Abstract

Exposure to high salinity can trigger acclimation in many plants. Such an adaptative response is greatly advantageous for plants and involves extensive reprogramming at the molecular level. Acclimation allows plants to survive in environments that are prone to increasing salinity. In this study, diploid and autotetraploid *Paulownia fortunei* seedlings were used to detect alterations in leaf proteins in plants under salt stress. Up to 152 differentially abundant proteins were identified by Multiplex run iTRAQ-based quantitative proteomic and LC-MS/MS methods. Bioinformatics analysis suggested that *P*. *fortunei* leaves reacted to salt stress through a combination of common responses, such as induced metabolism, signal transduction, and regulation of transcription. This study offers a better understanding of the mechanisms of salt tolerance in *P*. *fortunei* and provides a list of potential target genes that could be engineered for salt acclimation in plants, especially trees.

## Introduction

Salinization is one of the most adverse environmental stresses affecting plant growth and crop productivity in many areas of the globe, especially in already susceptible regions [1]. Plants have adapted dynamic responses to salt stress at the physiological, biochemical, and molecular levels to help in their survival [2].

In some plants, saltiness triggers the expression of a variety of genes that function in the plant’s salt response as well as in gene expression regulation and signal transduction to increase salt tolerance. Understanding how plants behave under such conditions and applying the knowledge to improve their performance in these situations is of interest to plant scientists. In recent decades, several advances have been made towards identifying potential salt stress-related genes that are capable of increasing the tolerance of plants to salt stresses [3, 4]. It was found that in this process different biochemical and molecular mechanisms act together in a complex regulatory network. These interactions are controlled by hormone signaling pathways that can induce or antagonize one another; in particular, the pathways that involve abscisic acid [5]. Transcription factors [6], kinase cascades [7], reactive oxygen species (ROS) [8], heat shock factors [9], and small RNAs [10] are key components of the molecular crosstalk in these pathways.

High-throughput genomic and transcriptomic sequencing techniques are powerful tools that have been used to gain comprehensive genomic information of the novelty and plasticity underlying the adaptation response of plants under salt stress. Many studies have focused on the transcriptional reprogramming of the related biological processes [11–13]. However, because most biological functions are carried out by proteins, and posttranscriptional regulation and posttranslational modifications can influence a system, quantitation of the proteins in a system is mandatory to understand the biological process and functions involved [14, 15]. Proteomics is a powerful tool for the identification of proteins and mechanisms involved in the salt stress response and tolerance [16–19]. Salt stress responsive proteins include those involved in basic metabolic pathways and lignification. Researches on drought stress in rice leaves suggested that 42 proteins, which were induced in mild drought stress, were completely reversed within 10 days of rewatering. Plants that have adapted to prolonged drought stress are capable of recovering more completely than unadapted plants after rewatering [20]. The concentration of proline, a compatible solute and also an osmoprotectant, was found to increase by up to 20-fold in some plants under severe drought conditions [21, 22]. Proline may protect or induce stress protective proteins and thus confer a protective effect. It was reported that severe salt stress inhibited the antioxidative enzymes catalase and peroxidase, and that their activity was maintained at significantly higher levels in the presence of proline in *Pancratium maritimum* [23].

Proteomics data for Paulownia species are particularly desirable because some of these species are of economic and forestry importance. Several transcriptome studies of Paulownia have been reported [24–27]. However, up to now, there is little Paulownia protein information in the public databases, despite the availability of large numbers of transcriptome unigenes. To investigate the salt stress response of the proteome in Paulownia, in this study, we conducted a systematic proteomic analysis during the plants’ acclimation to salt stress. Proteins from control and salt-treated *Paulownia fortunei* were identified and common proteins associated within this process were detected. A total of 152 proteins that responded to salt stress were identified by mass spectrometry. As a result, we established a comprehensive inventory of salinity-responsive proteins, many of which were involved in functions that included photosynthetic processes, ROS scavenging, signaling, and stress defense. We found that polyploidization affected the salt-responsive pattern, especially that of ion channel proteins. The autotetraploid *P*. *fortunei* had more salt-responsive proteins than the diploid *P*. *fortunei*. To our knowledge, this is the first reported proteome study on salt-treated Paulownia. This analysis provides a deeper insight of the acclimation process, which will help to understand the general stress response to saltiness and to identify pivotal proteins involved in plant stress responses.

## Materials and methods

### Plant material

All biological materials in this study were from the Institute of Paulownia, Henan Agricultural University, Zhengzhou, Henan Province, China.

Uniformly grown tissue cultured seedlings of diploid (PF2) and autotetraploid *P*. *fortunei* (PF4) were cultured in 100-mL triangular flasks on 1/2 MS medium for 30 days. After that the seedlings were clipped from the roots. Samples with the same height and crown size were then transferred into plastic pots containing the same amount of ordinary garden soil, one pot for each plant.

Prior to salt stress treatment, the plants were housed randomly in an outdoor nursery for 50 days. Twelve plants from each accession were selected, including three controls and nine plants for salt treatment. For salt treatment, the PF2 and PF4 seedlings in the three groups were treated with 0.4% NaCl solution (1 L) every two days for 15 days. The salt-treated PF2 and PF4 samples were named PFS2 and PFS4, respectively. The PF2 and PF4 seedlings in the control group were irrigated with tap water every two days for 15 days. Healthy leaves (second leaf from the apex) were harvested at 15 days, and equal numbers of leaves from each plant in each of the replicate groups were pooled to form the four samples. Terminal buds of 1.5 cm in length of the plantlets in the four treatment groups were sheared, immediately frozen in liquid nitrogen, and stored at −80°C.

### Protein extraction, digestion, and iTRAQ labeling

The PF2, PF4, PFS2, and PFS4 samples were ground into powder in liquid nitrogen, extracted with lysis buffer containing 1 mM PMSF and 2 mM EDTA. After that, proteins were extracted according to the method of Tang et al. [28]. Total protein was digested and iTRAQ-labelled according to Meng et al. [29]. Two biological replicates were set for each sample. Eight samples were labeled with iTRAQ tags as follows: PF2-1, 113; PF2-2, 117; PF4-1, 114, PF4-2, 118; PFS2-1, 119, PFS2-2, 115; PFS4-1, 116, PFS4-2, 121. The peptides were labeled with the isobaric tags and incubated at room temperature for 2 h. The labeled peptide mixtures were then pooled for strong cation exchange and dried by vacuum centrifugation.

### Strong cation exchange

Strong cation exchange chromatography was performed with a LC-20AB HPLC pump system. The iTRAQ-labeled peptide mixtures were reconstituted with 4 mL buffer A and loaded onto a 4.6 × 250 mm Ultremex SCX column containing 5-μm particles. The peptides were eluted at a flow rate of 1 mL min^−1^ with a gradient of buffer A for 10 min, 5–60% buffer B for 27 min, and 60–100% buffer B for 1 min. The system was then maintained at 100% buffer B for 1 min before equilibrating with buffer A for 10 min prior to the next injection. Elution was monitored by measuring the absorbance at 214 nm, and fractions were collected every 1 min. The eluted peptides were pooled into 20 fractions, desalted with a Strata X C18 column, and vacuum-dried.

### Liquid chromatography tandem-mass spectrometry (LC-MS/MS) analysis

Mass spectroscopy analysis was performed using an AB SCIEX TripleTOF™ 5600 mass spectrometer, coupled with an online micro-flow HPLC system as described in section 1.3. The peptides were separated using the method of Qiao et al. [30].

### iTRAQ protein identification and quantification

The acquired raw data files were converted into Mascot generic format files according the method of Lin et al. [31]. The Mascot 2.3.02 (Matrix Science, London, United Kingdom) was used for protein identification and quantification according the method of Guo et al. [32]. Protein identification was performed using the Mascot search engine against a Paulownia transcriptome database that contained 82,934 sequences. The sequencing data have been submitted to the Short Reads Archive under accession number SRP034738 [26]. To reduce the probability of false peptide identification, only peptides with significance scores in the 99% confidence interval greater than “identity” in a Mascot probability analysis were counted as identified. Each confident protein identification involved at least one unique peptide.

To reduce the probability of false peptide identification, only peptides with significance scores in the 99% confidence interval greater than “identity” in a Mascot probability analysis were counted as identified. Each confident protein identification involved at least one unique peptide.

For protein quantization, each protein was required to contain at least two unique peptides. The quantitative protein ratios were weighted and normalized by the median ratio in Mascot. The student’s T-test integrated into the Mascot software was used. Only the proteins with ratios>1.2or<0.84 and *p*-values<0.05 were considered as differentially abundant proteins (DAPs).

### Bioinformatics analysis

Functional analysis of the identified proteins was conducted using Gene Ontology (GO) annotations and the proteins were assigned terms under the biological process, molecular function, and cellular component categories. The proteins were further analyzed using the Clusters of Orthologous Groups (COG) of proteins database and the Kyoto Encyclopedia of Genes and Genomes (KEGG) database [33].

### RNA preparation and quantitative RT-PCR

In this analysis, the salt-treated PF2 samples were named PFS2-1 and PFS2-2, which corresponded to PF2 treated with salt for 15 days and 30 days, respectively. PFS4-1 and PFS4-2 were named in the same way. The RNA from these samples were extracted with Trizol (Invitrogen, CA, USA). The RNA samples from PF2, PF4, PFS2-1, PFS2-2, PFS4-1, and PFS4-2 were extracted with Trizol. The RNA was then precipitated with isopropanol. Purified and concentrated RNA was denatured and first-strand cDNAs for all the samples were synthesized using a PrimeScript RT reagent kit (Takara Biotechnology, Dalian, China). The genes encoding eight DAPs were selected for qRT-PCR analysis. Primers were shown in Table 1, 18S rDNA was selected as the reference gene. The method of qRT-PCR and relative expression calculation was described by Fan et al. [10].

**Table 1:** Primers of quantitative RT-PCR analysis of candidate DAP genes.

Unigene-ID	Potential gene function	Size (bp)	Up/down^a^	Primer sequence
CL2476.Contig2	strand cell wall invertase	1181	Up	CCCACCCTTTACTGATACAC
				ACAAGCCAAGTCATTTCTCC
CL1435.Contig2	chaperonin-60 alpha subunit, complete cds	2285	Down	ACCAAGAGTGAGACCAACAG
				TGAAGAGTAGGAGAGTGAACC
Unigene38438	UDP-glucuronate decarboxylase 1	1388	Up	CGAAGGACAACTTAAAGCAATGG
				GAAGCAGGACAAGCAAGATGG
Unigene35182	RSI3	1428	Up	AACACCTCCGCCGCTATG
				GCCAACCAGACTATCAAGATTCC
CL6922.Contig2	peroxidase	1402	Up	GCAGAATTGAAGGCTTGAGG
				GGAATGTGGACCCGAGAATAG
Unigene14040	stromal 70 kDa	992	Down	AGACTCCTCATCAACCTCATTC
				GCAGCCTACTATCATAACTAACG
Unigene31115	Photosystem I reaction center subunit II	237	Up	ACTGGAGTTGGGCAGAATATG
				TCACAAATCATACACTTGCTTCC
CL10694.Contig2	2-oxoglutarate dehydrogenase	405	Down	CGGCTGTTGTTGCTTGTG
				AACCCATACATTCTCCAAACG

^a^ Up or down regulation indicated the expression in both the PFS4 *vs*. PF4 and the PFS2 *vs*. PF2 comparisons.

## Results

### Proteomics characterization

**Fig 1:**
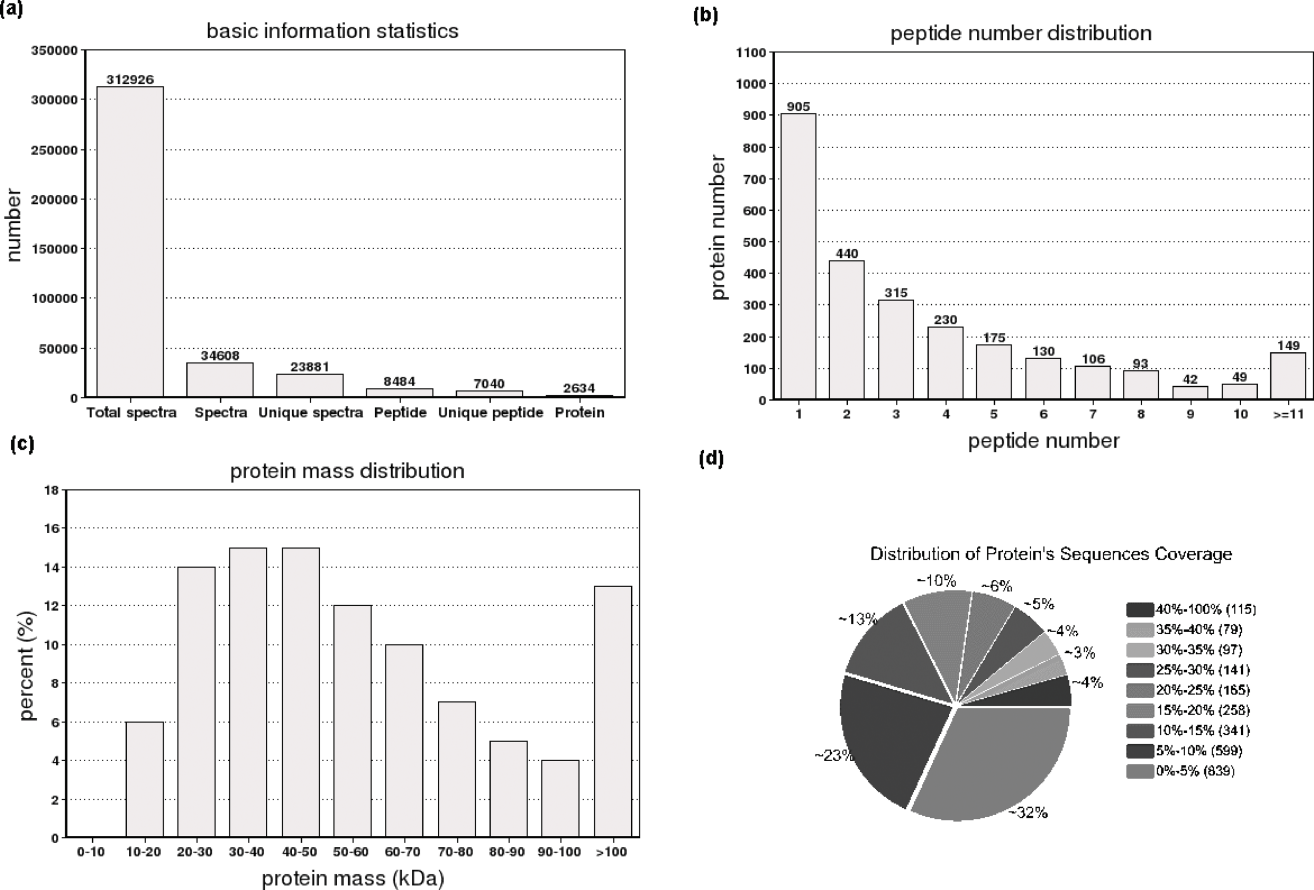
Summary of the iTRAQ-based *Paulownia fortunei* proteome. (a) Spectra, peptides and proteins that were identified by searching against the *Paulownia fortunei* transcriptome database; (b) Number of peptides that match proteins using MASCOT; (c) Distribution of the proteins that were identified among different molecular weights; (d) Coverage of the proteins by the identified peptides.

Proteins were extracted from control and salt-treated diploid and autotetraploid *P*. *fortunei* (PF2, PFS2, PF4, and PFS4) seedling leaves and a total of 312,926 spectra were generated. 34,608 spectra that matched known spectra were identified by the Mascot software. Among them, 23,881 unique spectra were matched to 7,040 unique peptides and 2,634 proteins (Fig 1A), approximately 66% of which consisted of at least two of the unique peptides (Fig 1B). The vast majority of these proteins were larger than 10 kDa, although their molecular weights covered a wide range (Fig 1C). Most of the identified proteins had good peptide coverage; 45% had more than 10% sequence coverage, and 22% had 20% sequence coverage (Fig 1D).

**Fig 2:**
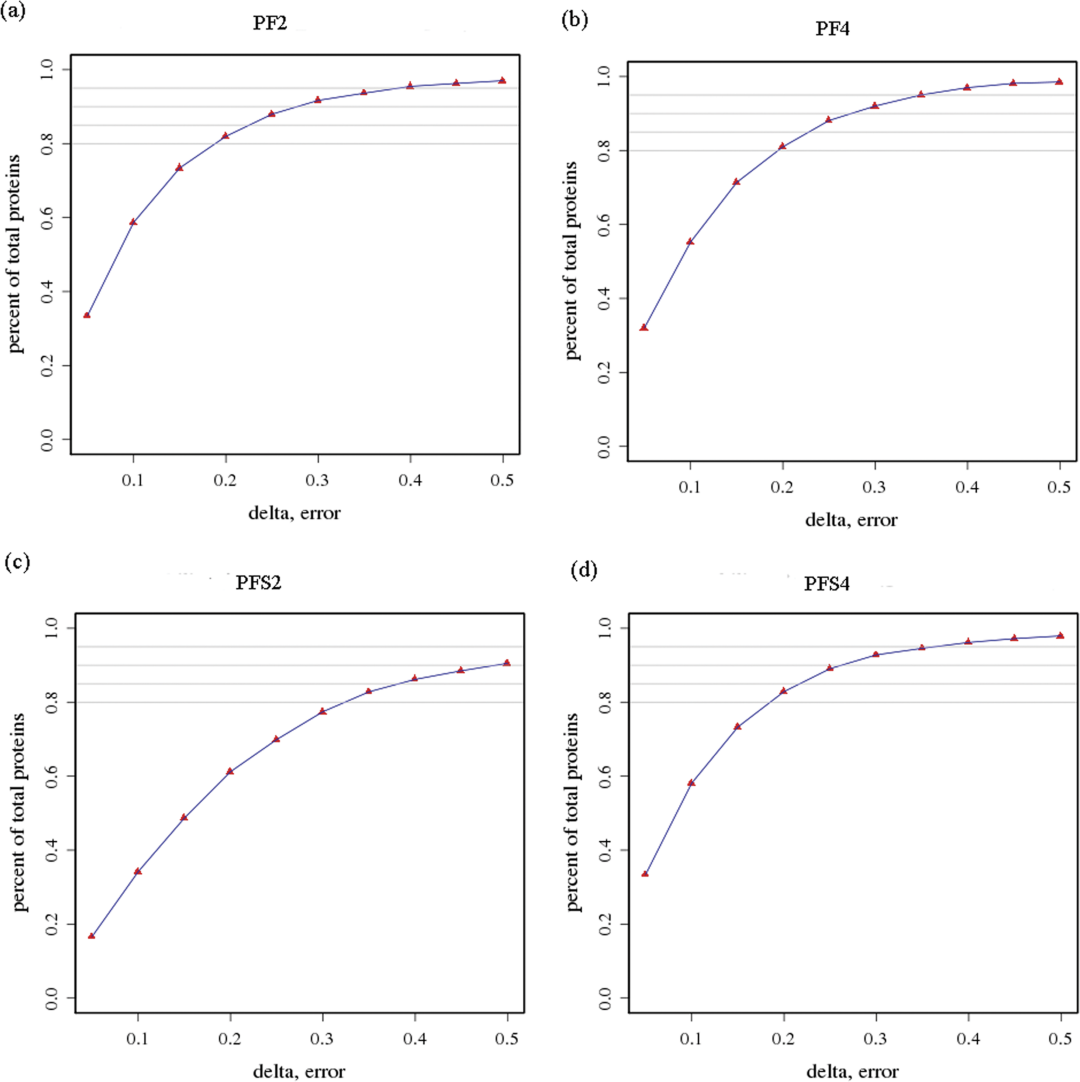
Repeatability of the expression of duplicate samples. (a) PF2; (b) PF4; (c) PFS2; (d) PFS4. X-axis represents the difference of the quantitative ratios between the first and the second biological repeats of the two samples. The right y-axis represents the cumulative percentage between the proteins of a certain range and the quantitative proteins, while the left y-axis represents the number of total protein in a certain range.

To avoid identification omissions, less than 2 ppm were confined as the database search strategy for the peptide matching error. The reproducibility of the proteomic analysis was shown in Fig 2. These results indicate that our proteomics analyses were reliable.

**Fig 3:**
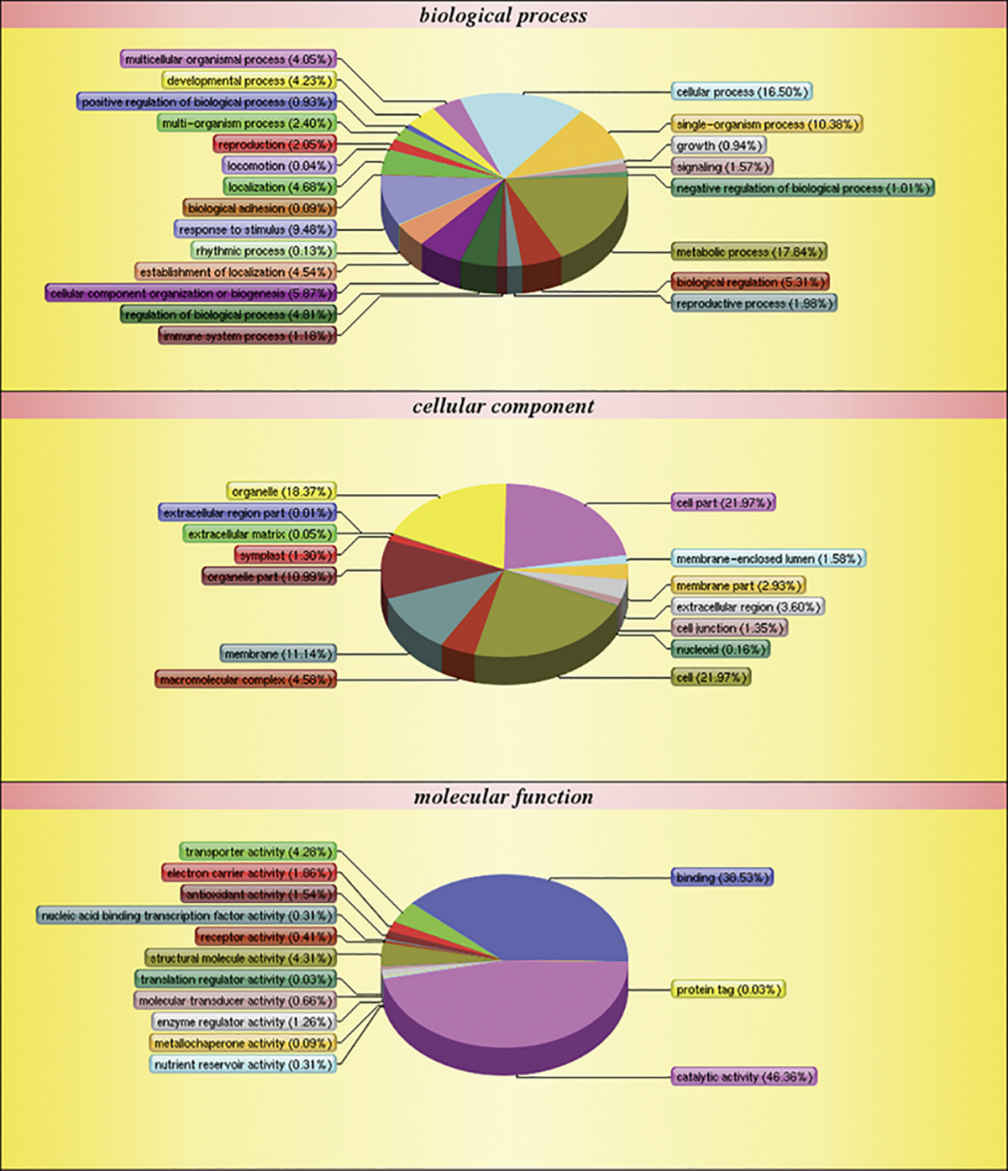
Gene Ontology classification of distinct proteins that were detected in *Paulownia fortunei* leaves.

To carry out a functional analysis, we assigned gene ontology (GO) terms to all the quantitated proteins. Under the biological process category, metabolic process (1,305) and cellular process (1,003) were the most represented groups; under the cellular component category, cell (1,040) and cell part (1,040) were the two largest groups; and under molecular function category, catalytic activity (1,153) and binding (900) were most represented (Fig 3). These results indicate that the identified proteins are involved in almost every aspect of *P*. *fortunei* metabolism.

**Fig 4:**
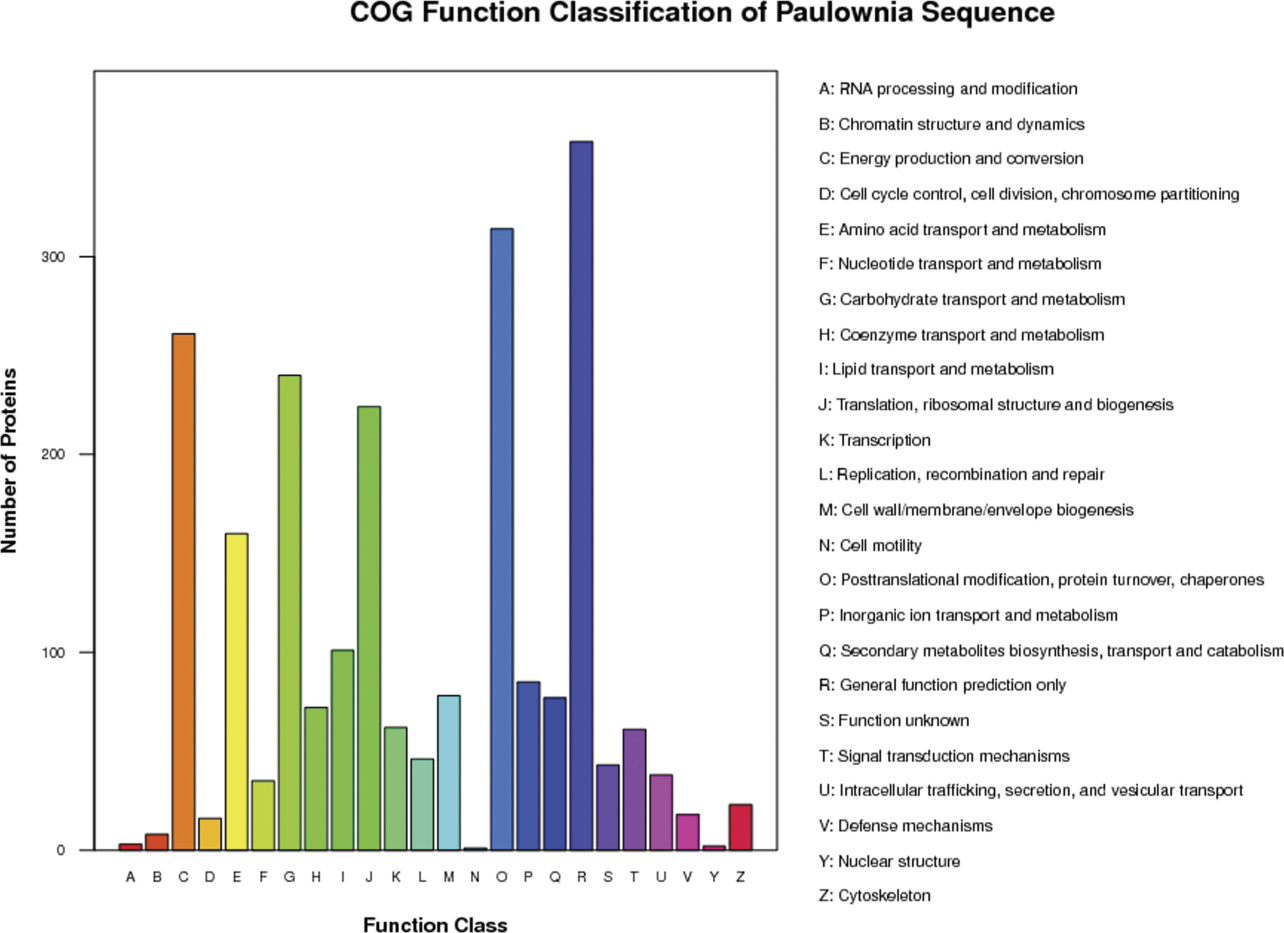
Classification of the clusters of orthologous groups function classification of distinct proteins that were detected in *Paulownia fortunei* leaves.

To further understand the functions of the 2,634 proteins, they were assigned to 24 categories in the Clusters of Orthologous Groups (COG) database. The most highly represented functional categories were “general function prediction only” and “posttranslational modification, protein turnover, chaperones”, which represented approximately 14% and 12% of the identified proteins, respectively (Fig 4).

The annotated proteins were mapped onto 128 Kyoto Encyclopedia of Genes and Genomes (KEGG) pathways (S1 Table). Among them, “metabolic pathways” (830) was significantly more highly represented than the other pathways, followed by “biosynthesis of secondary metabolites” (452), and “carbon metabolism” (192). Based on these analyses, we concluded that most of the mapped proteins may affect cellular component biogenesis, posttranslational modification, protein turnover, and response to stimulus.

### Analysis of the differentially abundant proteins related to salt stress in the autotetraploid

Differentially abundant proteins (DAPs) were defined as the proteins that showed a >1.2-fold or <0.84-fold change in relative abundance and a *p*-value <0.05. A total of 767 DAPs were identified in the PF4 *vs*. PF2 comparison; 360 were more abundant and 407 were less abundant. A total of 916 DAPs were identified in the PFS2 *vs*. PF2 comparison; 488 were more abundant and 428 were less abundant. A total of 712 DAPs were identified in the PFS4 *vs*. PF4 comparison; 390 were more abundant and 322 were less abundant. A total of 693 DAPs were identified in the PFS4 *vs*. PFS2 comparison; 399 were more abundant and 294 were less abundant. We identified the common DAPs between the PFS2 *vs*. PF2 and PFS4 *vs*. PF4 comparisons to detect DAPs that were involved in salt stress in both diploid and autotetraploid plants. The list of these common DAPs was then aligned with the list of DAPs from the PF4 *vs*. PF2 comparison and the DAPs that were represented in the two lists were identified as DAPs that were involved in salt stress only in the autotetraploid plants (S2 Table).

**Fig 5:**
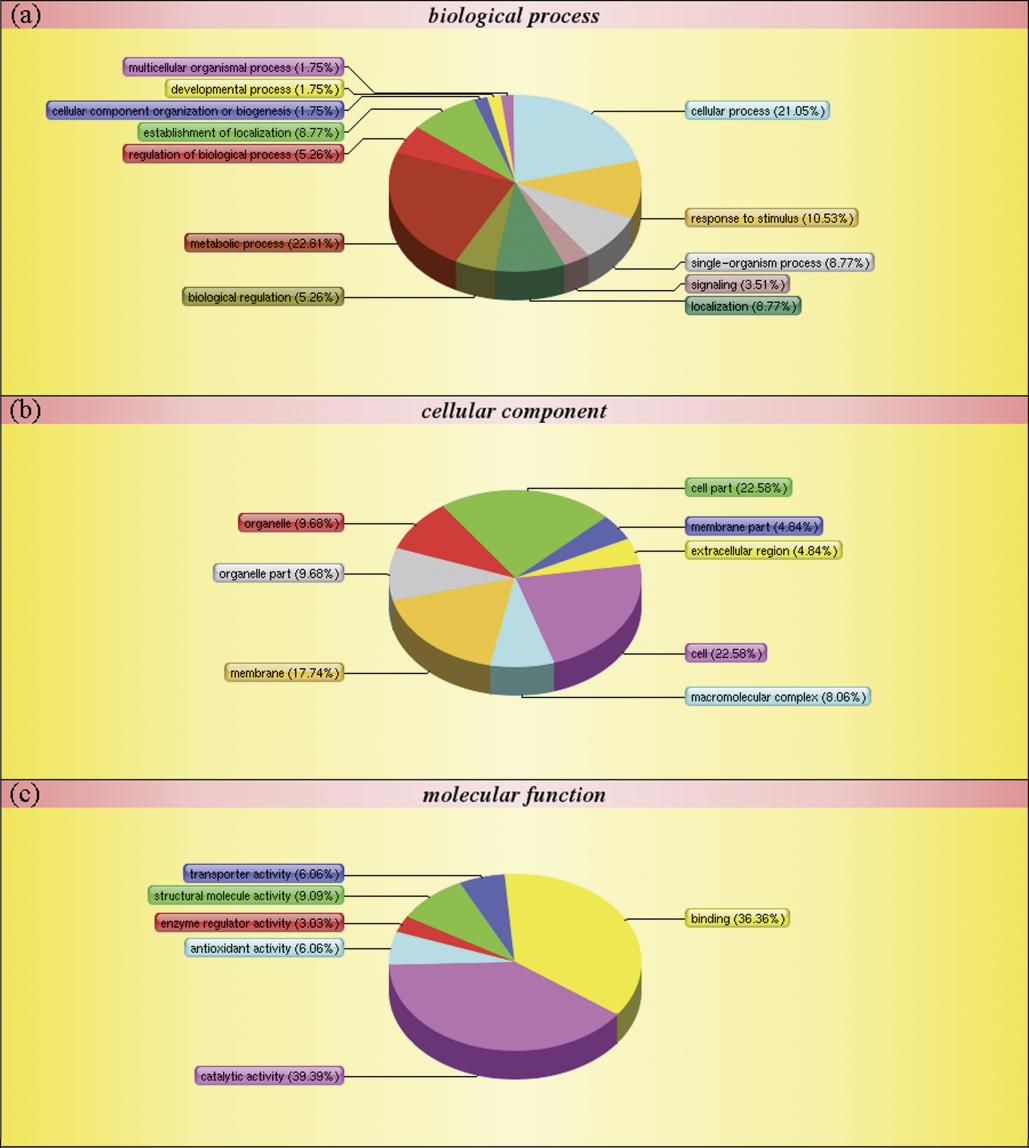
Gene Ontology classification of differentially abundant proteins that were detected in *Paulownia fortunei* leaves.

The autotetraploid-specific salt stress DAPs were then analyzed using the GO, COG, and KEGG databases. A total of 152 DAPs were assigned to 26 functional groups under the three main GO categories (Fig 5 and S3 Table).

Under biological process, metabolic process and cellular process were the most represented groups; under cellular component, cell and cell part were the two largest groups; and under molecular function, catalytic activity and binding were the most represented. Interestingly, the GO classifications for the DAPs were similar to the classifications for all the identified proteins. The most enriched GO terms under biological process, cellular component, and molecular function were the same for all the proteins and for the autotetraploid-specific salt stress DAPs subset.

To predict and classify the possible functions of the DAPs, we assigned them to COG categories. Based on their sequence homology with known proteins in GenBank, 28 DAPs (1.06% of all the proteins) were divided into 11 categories (Table 2). The “posttranslational modification, protein turnover, chaperones” category contained five proteins and was the largest, followed by “energy production and conversion” (4), “translation, ribosomal structure and biogenesis” (4), “carbohydrate transport and metabolism” (4), “general function prediction only” (3) (S1 Fig).

**Table 2:** Statistics of the functional categories of the DAPs that were detected in *P*. *fortunei* leaves.

Code	Functional-Categories	DAP-Number
C	Energy production and conversion	4
E	Amino acid transport and metabolism	2
G	Carbohydrate transport and metabolism	4
I	Lipid transport and metabolism	1
J	Translation, ribosomal structure and biogenesis	4
M	Cell wall/membrane/envelope biogenesis	2
O	Posttranslational modification, protein turnover, chaperones	5
P	Inorganic ion transport and metabolism	1
Q	Secondary metabolites biosynthesis, transport and catabolism	1
R	General function prediction only	3
S	Function unknown	1

The DAPs were mapped to 22 KEGG metabolic pathways (S4 Table); among them, “metabolic pathways” were highly enriched, followed by “biosynthesis of secondary metabolites”, “starch and sucrose metabolism”, and “ribosome”.

### Confirmation of DAPs by qRT-PCR

**Fig 6:**
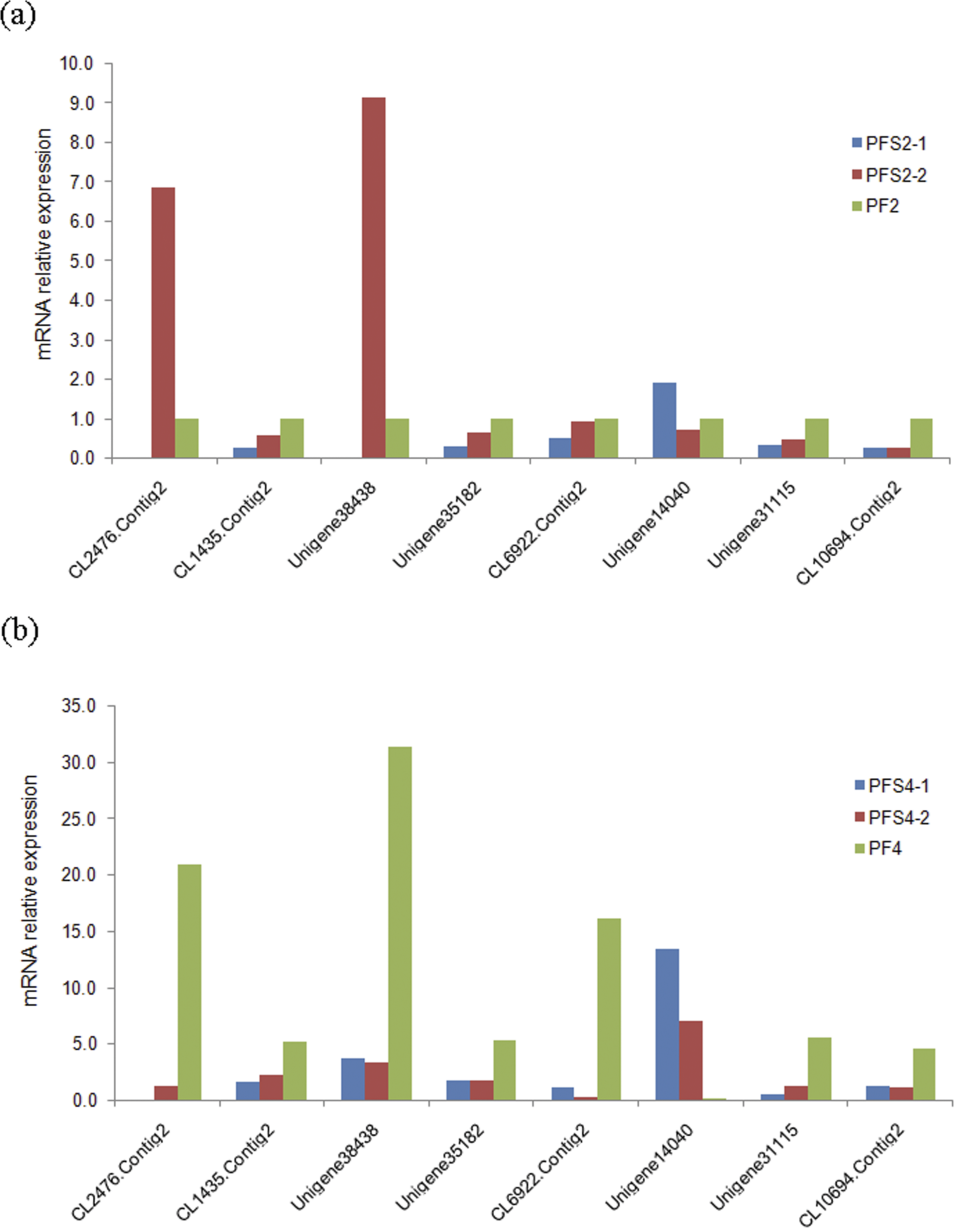
Quantitative RT-PCR analysis of *Paulownia fortunei* candidate proteins. The 18S rRNA of Paulownia was chosen as an internal reference gene for normalization. CL2476.Contig2: strand cell wall invertase; CL1435.Contig2: chaperonin-60 alpha subunit; Unigene38438: UDP-glucuronate decarboxylase 1; Unigene35182: RSI3; CL6922.Contig2: peroxidase; Unigene14040: stromal 70 kDa; Unigene31115: Photosystem I reaction center subunit II; CL10694.Contig2: 2-oxoglutarate dehydrogenase.

To confirm the DAPs identified by the iTRAQ analysis, qRT-PCR was performed to detect the transcript expression levels of the corresponding genes. The qRT-PCR results showed that in the PFS2 *vs*. PF2 and PFS4 *vs*. PF4 comparisons, the expression level of the gene corresponding to one of the DAPs was consistent with the iTRAQ LC-MS/MS analysis results, while the expression levels of the genes corresponding to three DAPs showed opposite trends (Fig 6). Interestingly, in the PFS2 *vs*. PF2 comparison, the expression levels of the genes corresponding to six DAPs displayed the same trends in the qRT-PCR and iTRAQ analysis and only two displayed opposite trends, while in the PFS4 *vs*. PF4 comparison, the expression levels of the genes corresponding to seven DAPs displayed opposite trends and only one displayed the same trend. These discrepancy may be attributed to posttranscriptional and posttranslational regulatory processes [34].

## Discussion

Salt stress is expected to become more severe and has been recognized as a major threat to global plants in the future. Although several approaches have been advocated to address this problem, due to the genetic and physiological complexity of the mechanisms involved, limited success has been achieved in attempts to breed salt-tolerant plants [35]. Thus, understanding the molecular mechanisms of salt tolerance of plants has been a focus of much research. An enhanced understanding of the associated genes and molecular mechanisms involved, should make it easier to breed salt-tolerant plants [36]. Altered abundance levels of proteins due to changes in the expressions of many genes occur when plants are subjected to salt stress. These changes may confer susceptibility or protection to salt stress. These changes may confer susceptibility or protection to salt stress. To identify the functions of individual genes that control traits associated with tolerance to salt stress, iTRAQ techniques are used to identify candidate protein for salt stress tolerance in many plants [37, 38]. In this study, to detect variations in the responses of diploid and autotetraploid *P*. *fortunei* to salt stress, we employed a proteomic approach on salt-treated and normal diploid and autotetraploid *P*. *fortunei* seedlings.

Cell expansion and growth are the first processes to decline when a plant is subjected to salt stress. After that, with more severe salt stress, photosynthesis is adversely affected. Then, a reduction in hydration and increase in ROS can damage cellular membranes and proteins [35]. Plants have developed various mechanisms to overcome such damage, for example, osmotic pressure is often changed during salt stress in plants due to the accumulation of solutes [39] and alterations in the structure of aquaporins or ion channel proteins. Among this process, proline and glycine betaine are the most commonly accumulated compounds in plants in response to environmental stresses [40]. At the same time, soluble sugars can act as moderators of osmotic pressure, in general, salt stress can alter the concentrations of soluble sugars, such as glucose, sucrose, and fructose [41]. However, in severe dehydration conditions, soluble sugars act opposite with their former alteration [42]. Soluble sugars can act not only as moderators of osmotic pressure, but also as signal transductors in salt stress [41] when they may interact with plant hormones to regulate the expression of other genes, such as those involved in photosynthetic metabolism [43]. In our study, sugar transporter as the DAP was up-regulated in the salt stress in the leaf of diploid and autotetraploid *P*. *fortunei*. It seems that the accumulation of sugar was closely related to accumulation of sugar transporter genes. In the salt stress wheat, two sugar transporter genes *SuT4* and *SuT5* were up-regulated, which had greater inhibitory effects on plant growth [36]. In addition, changes of osmotic pressure also as signal transductors may interact with plant hormones to regulate the expression of other genes, such as those involved in photosynthetic process, which further affected plant growth [43, 44]. Therefore, changes of osmotic pressure in leaves may be one of the important regulation pathway in the diploid and autotetraploid *P*. *fortunei* under salt stress.

Leaf Na^+^ and the K^+^/Na^+^ discrimination ratio, were proved to be important in salt tolerance in plants[45]. A series of K^+^/Na^+^ channel proteins, Na^+^/H^+^ and Ca^2+^/H^+^ antiporters, and enzymes involved in the transport of ions were found to be altered in autotetraploid and salt-treated Paulownia, indicating that the proton gradient was important in the salt tolerance process [27]. Similarly, a gene encoding vacuolar ATPase subunit B in a salt-sensitive barley cultivar was identified by differential display-reverse transcriptase PCR (DDRT-PCR) [46]. In roots, the ATPase can produce a proton gradient that can drive the uptake of Na^+^ and Ca^2+^ by Na^+^/H^+^ and Ca^2+^/H^+^ antiporters, respectively. Thus, it is supposed to be involved in detaining of Ca^2+^ and Na^+^ ions into the vacuole [47]. Genetic manipulation to add expression of K^+^/Na^+^ channel proteins, Na^+^/H^+^ and Ca^2+^/H^+^ antiporters, and enzymes involved in the transport of ions so as to increase osmoprotectant flux could be a good method to acclimate plants to salt stress [48]. Overall, the mechanisms of the Paulownia response to salt stress can be divided into two main types: minimizing the entry of salt into Paulownia plants, and minimizing the concentration of salt in the cytoplasm. The DAP, ATP synthase delta chain was up-regulated in the salt stress paulownia, play important role in stabilization of thylakoid protein composition, it was reported that knock-out of the genes coding for ATP synthase delta chain in Arabidopsis could affect the integrity of the thylakoid membrane, which further affect electron and proton transport across the thylakoid membrane, resulting in the reduction of the synthesis of NADPH and ATP [49].on the contrary, the up-regulated ATP synthase delta chain in Paulownia may enhance the ATP production and increase the activity of transport of ions to response salt stress.

In a previous study, we examined the differences between drought-treated diploid and autotetraploid *P*. *fortunei* using RNA sequencing [50]. The differentially expressed genes that we found in that study do not overlap well with the DAPs detected in this study. There may be several reasons for this discrepancy. Firstly, the plant response and acclimate bioprocesses under salt stress and under drought stress will not be identical. For example, Oztur et al. [51] reported that in barley, the expression of 5% of the genes were changed under salinity stress, while the expression of 15% of the genes were changed under drought stress. Secondly, posttranscriptional regulation and posttranslational modifications influence translational efficiency, which could lead to discordance between mRNA expression levels and protein abundance. Such discordance had been widely reported; for example, by Lan et al. in Arabidopsis roots [52].

## Conclusion

Dynamic changes of proteins abundance in the leaf of diploid and autotetraploid *P*. *fortunei* were investigated by iTRAQ analysis. A total of 2,634 proteins were identified. After analysis of the differentially abundant proteins, 152 autotetraploid-specific DAPs were obtained. The key metabolic pathways of DAP enriched pathways were detected. they were assigned to 26 GO functional groups and mapped to 22 KEGG metabolic pathways. The up-regulated sugar transporter might promote the accumulation of soluble sugars which might increase changes of osmotic pressure, and the up-regulated ATP synthase delta in ATP production increased the activity of transport of ions which may for proton gradient to response the salt stress. Currently, based on the fact of discordance between mRNA expression levels and protein abundance, we have only a limited understanding the roles of these proteins in the leaf of diploid and autotetraploid *P*. *fortunei* under salt stress, further research may focus on the functional genomic of the candidate proteins identified by our proteomic investigation.

## Supporting information

S1 Table KEGG pathway analysis of all identified proteins for *Paulownia fortunei*. (XLSX)

S2 Table Differentially abundant proteins identified in salt treated *Paulownia fortunei*. (XLSX)

S3 Table Gene Ontology analysis of differentially abundant proteins in salt treated *Paulownia fortunei*. (XLS)

S4 Table KEGG analysis of the identified differentially abundant proteins in salt treated *Paulownia fortunei*. (XLSX)

S1 Fig Classification of the clusters of orthologous groups for the Itraq-based proteotome of *Paulownia fortunei*. (TIF)
