# Supplementary figures and images for: Dissecting the proteome dynamics of the salt stress induced changes in the leaf of diploid and autotetraploid Paulownia fortunei
Source: PLoS One. 2017 Jul 27;12(7):e0181937. doi: 10.1371/journal.pone.0181937 (PMC5531653; doi:10.1371/journal.pone.0181937)

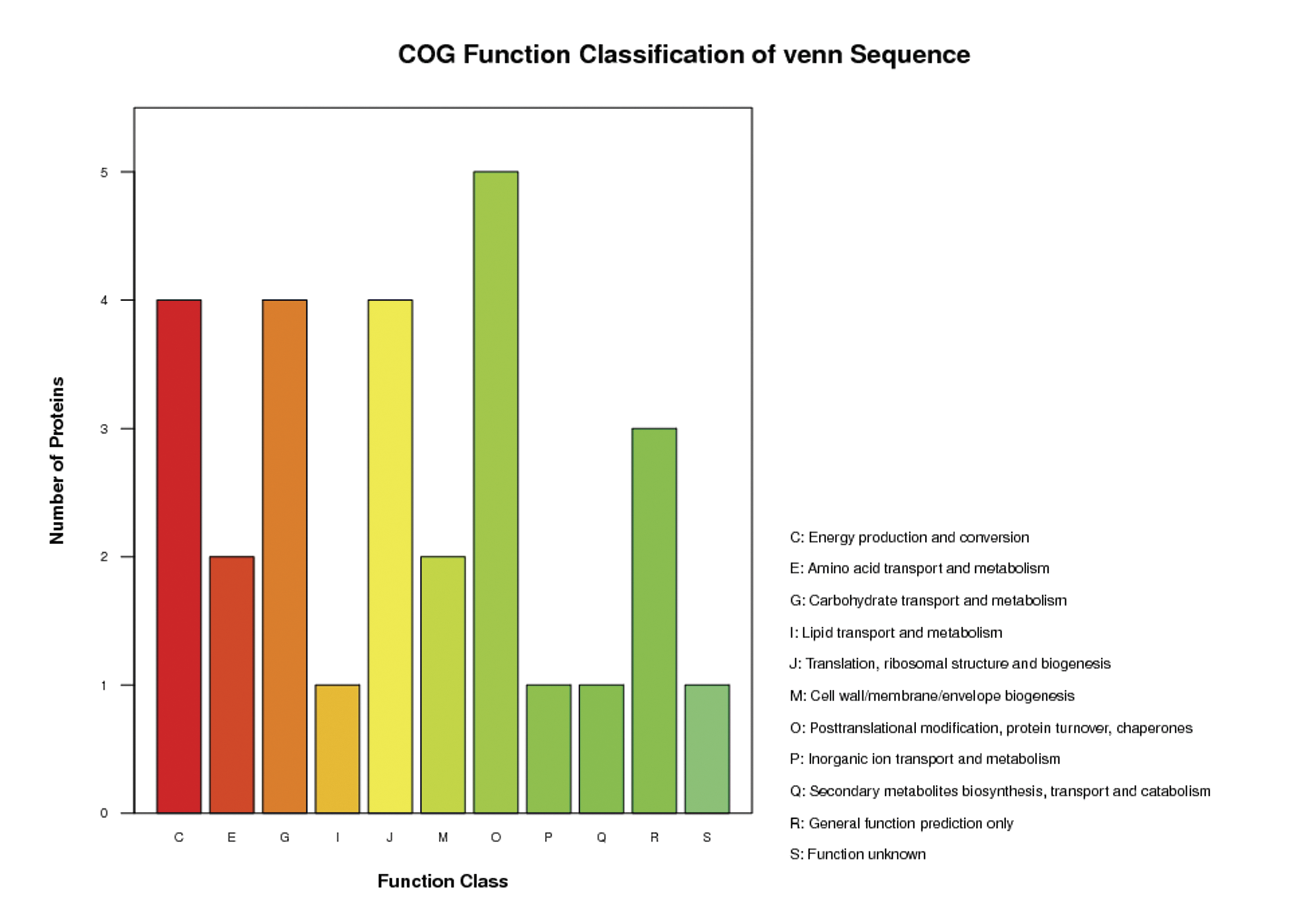

Supplement: S1 Fig — (TIF) [file pone.0181937.s005.tif]
